# Supplementary material for: Manifestations of Liver Impairment and the Effects of MH-76, a Non-Quinazoline α1-Adrenoceptor Antagonist, and Prazosin on Liver Tissue in Fructose-Induced Metabolic Syndrome
Source: Metabolites. 2023 Nov 3;13(11):1130. doi: 10.3390/metabo13111130 (PMC10672990; doi:10.3390/metabo13111130)
Supplement: Supplementary file 1 [file metabolites-13-01130-s001.zip › metabolites-2651772-supplementary.pdf]

Supplementary materials

Table S1. Systolic blood pressure and fasting glycemia at the week 12 in fructose-fed rats.

	**Systolic blood pressure (mmHg)**	**Fasting glycemia (mg/dl)**
Control	122.3 ± 3.5	79.0 ± 7.9
Fructose	138.4 ± 10.2 ***	100.8 ± 10.7 *
Fructose+MH-76	139.3 ± 8.3 ***	98.1 ± 7.6*
Fructose+Prazosin	135.6 ± 3.2 **	100.3 ± 20.8*

Data are presented as means ± SD, n = 7-8, *p < 0.05, **p < 0.01, ***p <0.001 vs Control, One-way ANOVA, post hoc Tukey test.

Table S2. Selected components of the metabolic syndrome of Control, Fructose, Fructose+MH-76, and Fructose+Prazosin- treated rats at the end of experimental period (week 18)

	**Abdominal fat weight (mg/g body wt)**	**Systolic blood pressure (mmHg)**	**Fasting glycemia (mg/dl)**	**HOMA-IR**	**Fasting triglycerides (mmol/l)**
Control	41.1 ± 5.0	119.4 ± 2.9	100.8 ± 10.3	1.75 ± 0.5	1.9 ± 0.5
Fructose	58.9 ± 6.4***	137.9 ± 5.6**	132.3± 8.4***	5.3 ±3.9*	3.7 ±0.8***
Fructose+MH-76	36.2 ± 6.8####	120.8 ± 17.4#	89.5 ± 16.8####	1.6 ± 0.6#	1.9 ± 0.5###
Fructose+Prazosin	57.7 ± 9.0***	115.4 ±10.6###	107.1 ± 13.5##	6.5 ± 2.0**	2.6 ± 1.1

Data are presented as means ± SD, n = 7-8, *p < 0.05, **p < 0.01, ***p < 0.001, ****p < 0.0001 vs Control, #p < 0.05, ##p < 0.01, ###p < 0.001, ####p < 0.0001 versus Fructose, One-way ANOVA, post hoc Tukey test.

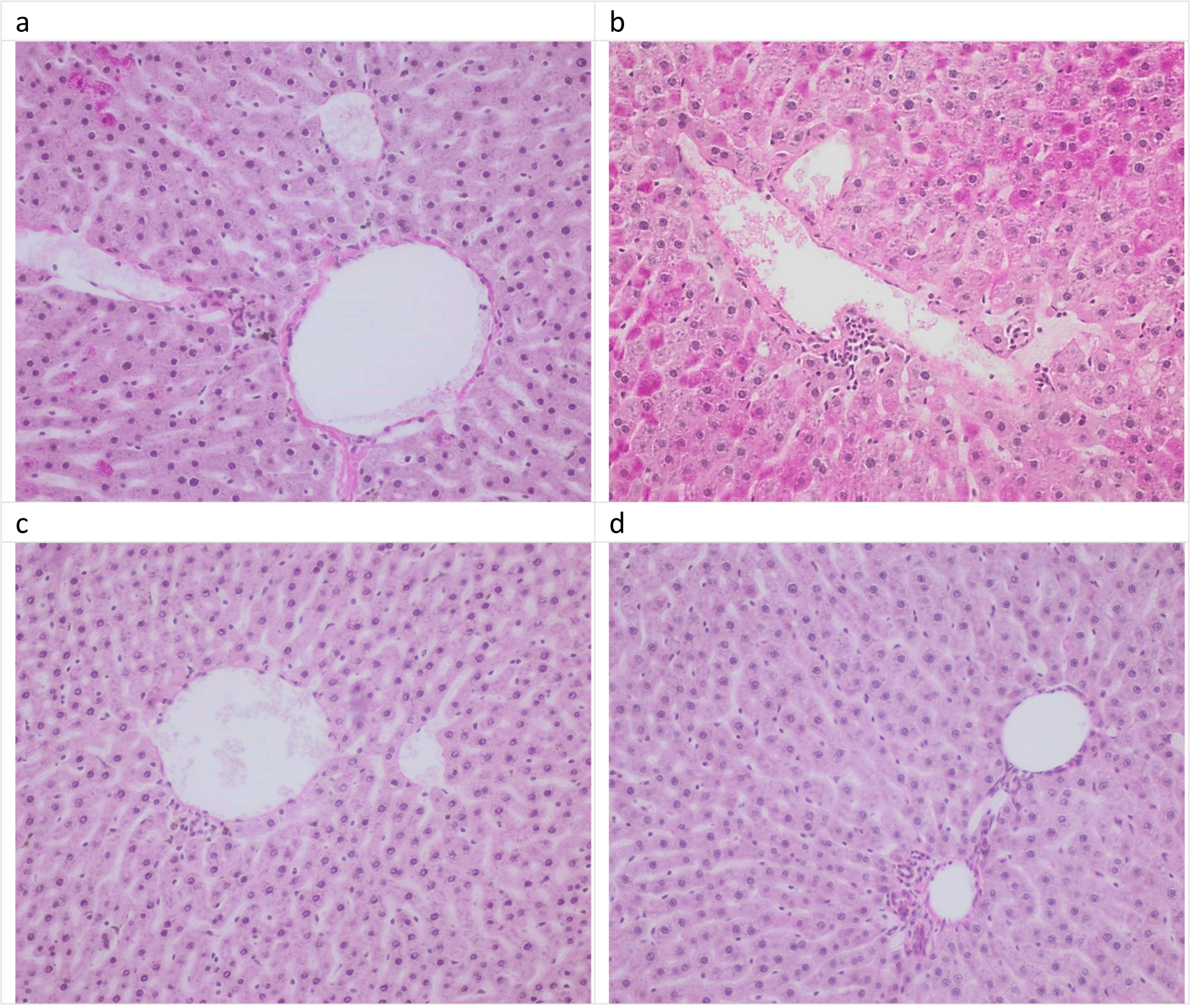

Figure S1. Lobular structure regularity and sinusoidal irregularity; **(a)** Control group, **(b)** Fructose, **(c)** Fructose+MH-76, (**d**) Fructose+Prazosin. H-E staining; Original magnification: objective 40x.

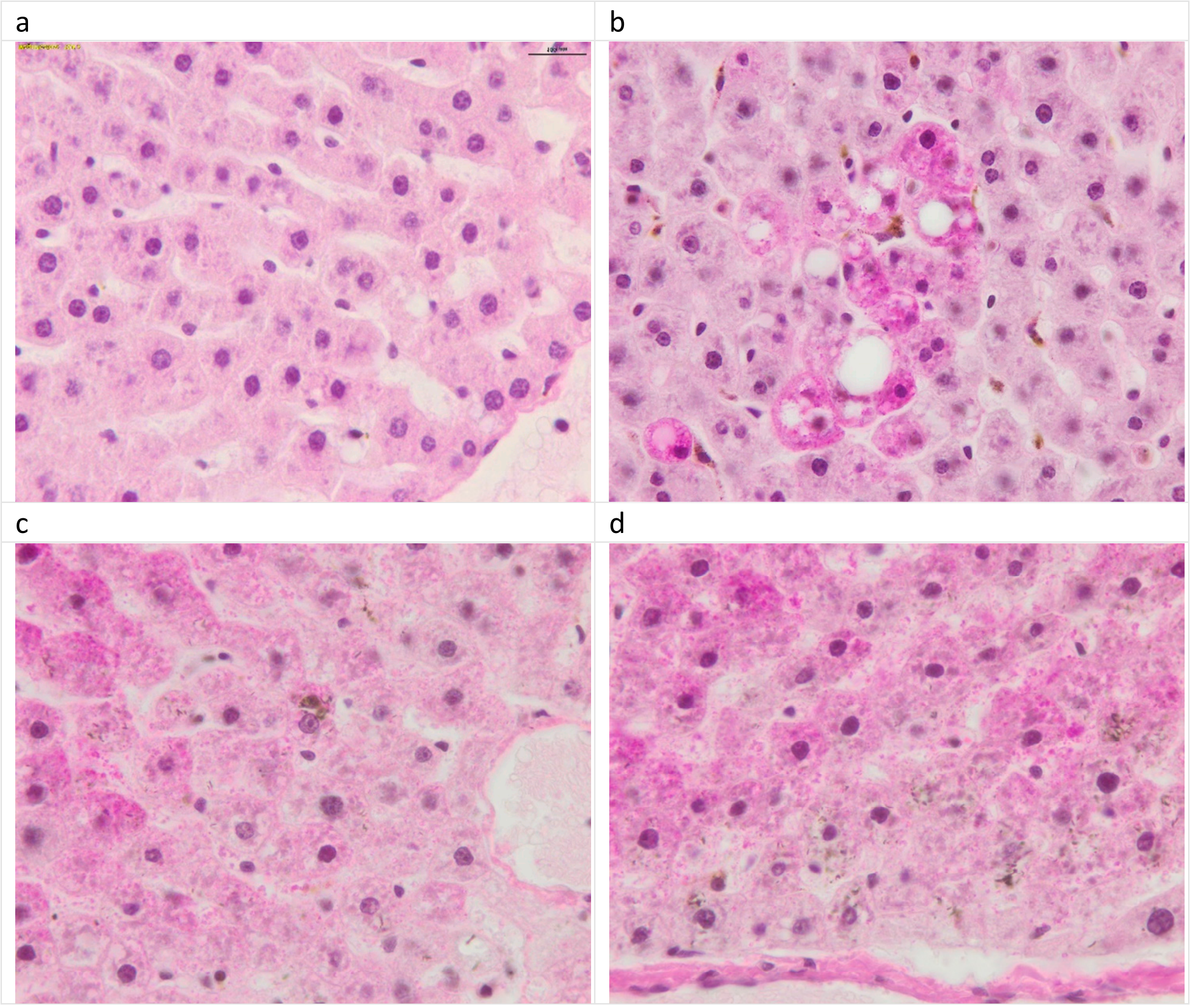

Figure S2. Fructose group: occasional focal inflammation with ballooning and necrotic cells, and Mallory-Denk bodies in comparison to other groups; **(a)** Control group, **(b)** Fructose, **(c)** Fructose+MH-76, (**d**) Fructose+Prazosin. H-E staining; Original magnification: objective 100x.

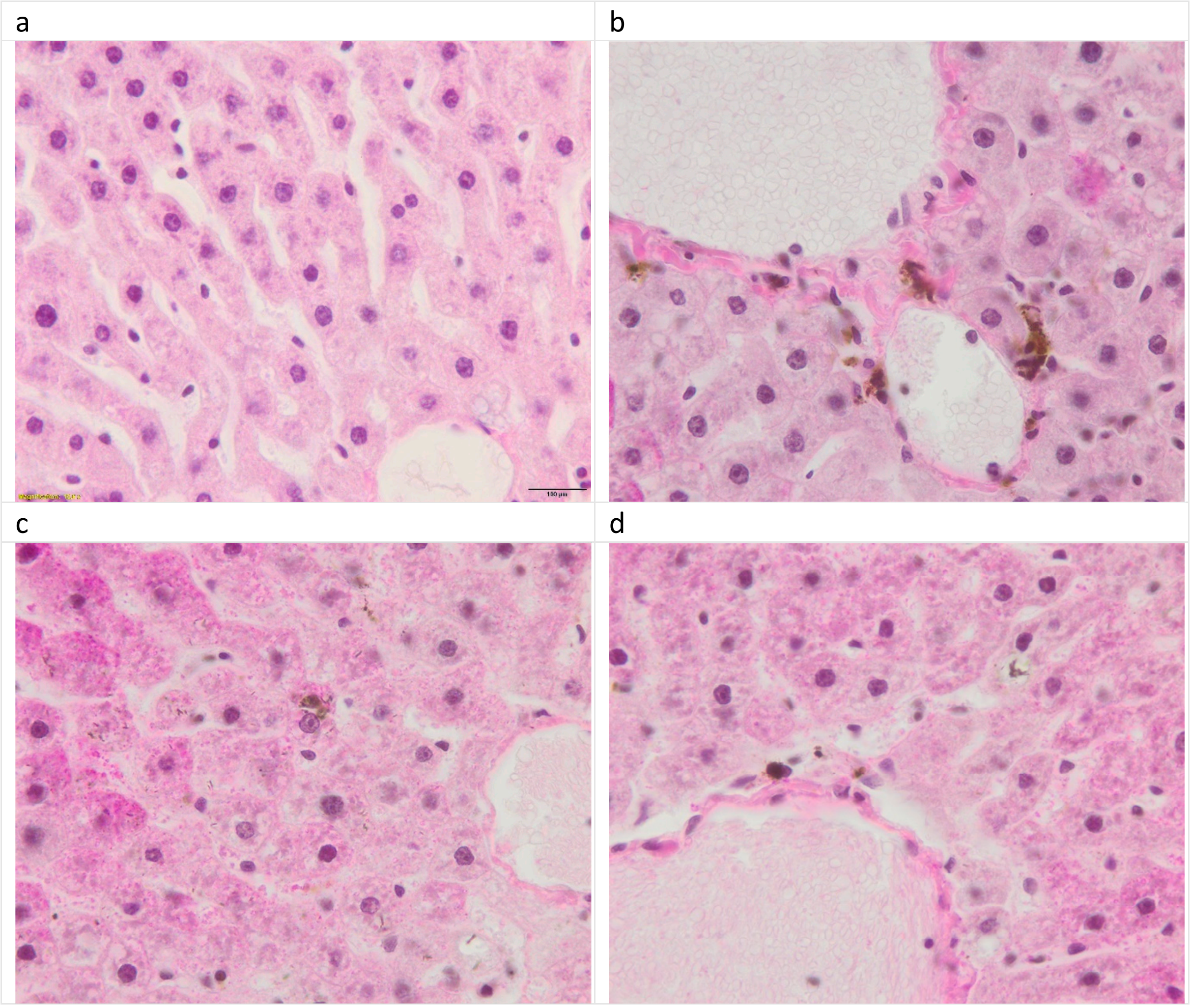

Figure S3. Kupfer cell accumulation in Fructose group in comparison to other groups; **(a)** Control group, **(b)** Fructose, **(c)** Fructose+MH-76, **(d)** Fructose+Prazosin. H-E staining; Original magnification: objective 100x.

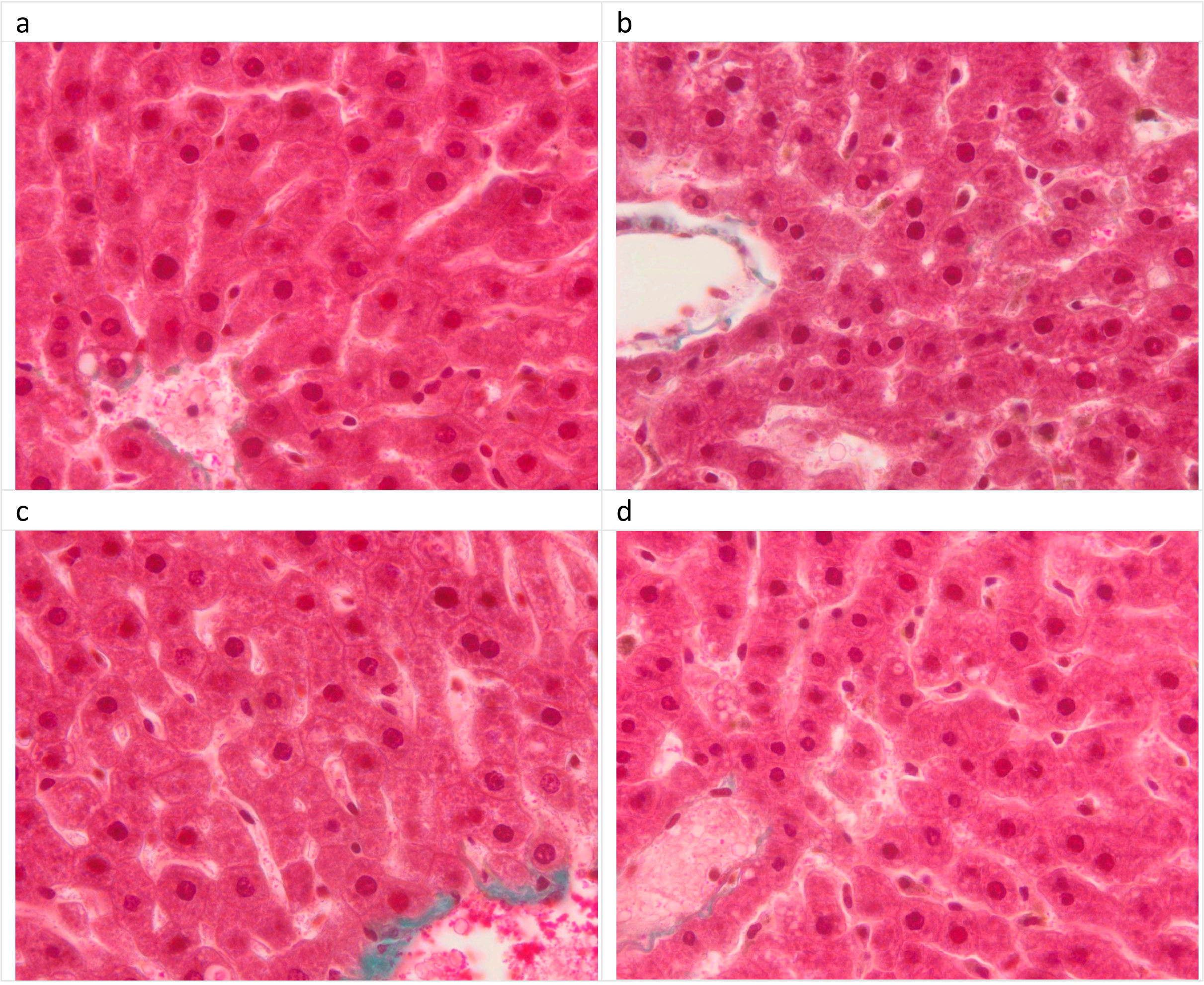

Figure S4. Micro- and macrosteatosis in Fructose group in comparison to other groups; **(a)** Control group, **(b)** Fructose, **(c)** Fructose+MH-76, (**d**) Fructose+Prazosin. H-E staining; Original magnification: objective 100x.

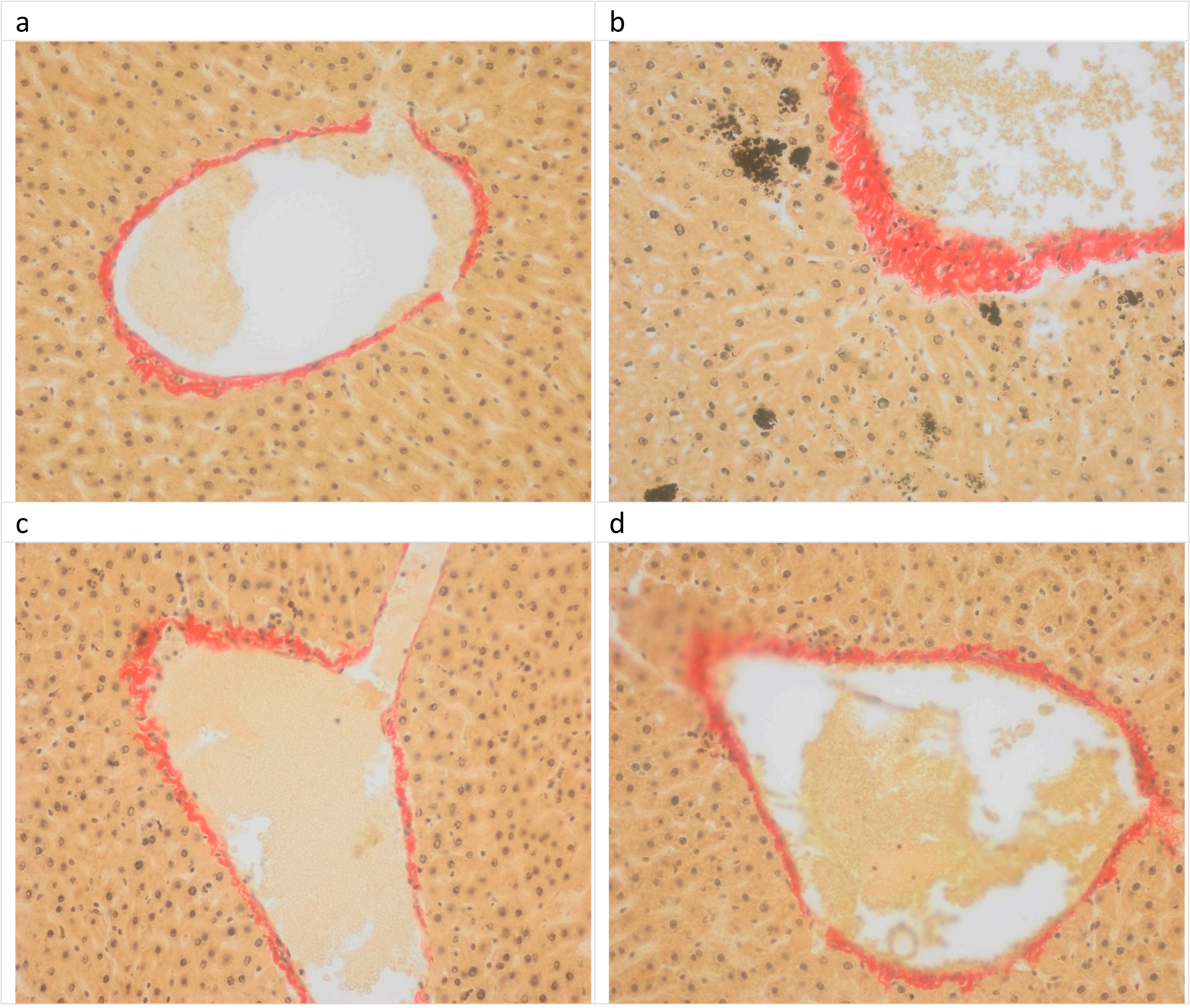

Figure S5. Pigment deposits within hepatocytes clustered near central vein (but occasionally has a panlobular distribution) and within Kupffer cells in sinusoids (here less con-spicuous) in Fructose group in comparison to other groups; **(a)** Control group, **(b)** Fructose, **(c)** Fructose+MH-76, **(d)** Fructose+Prazosin. H-E staining; Original magnification: objective 100x.
